# Cancer screening and breast cancer family history in Spanish-speaking Hispanic/Latina women in California

**DOI:** 10.3389/fonc.2022.1087022

**Published:** 2023-01-06

**Authors:** Lizeth I. Tamayo, Fabian Perez, Angelica Perez, Miriam Hernandez, Alejandra Martinez, Xiaosong Huang, Valentina A. Zavala, Elad Ziv, Susan L. Neuhausen, Luis G. Carvajal-Carmona, Ysabel Duron, Laura Fejerman

**Affiliations:** ^1^Department of Public Health Sciences, The University of Chicago, Chicago, IL, United States; ^2^Department of Public Health Sciences, University of California, Davis, Davis, CA, United States; ^3^Vision y Compromiso, Los Angeles, CA, United States; ^4^Promoters for Better Health, Pomona, CA, United States; ^5^Department of General Internal Medicine, University of California, San Francisco, San Francisco, CA, United States; ^6^Department of Population Sciences, Beckman Research Institute of City of Hope, Duarte, CA, United States; ^7^Department of Biochemistry and Molecular Medicine, University of California, Davis, Davis, CA, United States; ^8^Comprehensive Cancer Center, University of California Davis, Sacramento, CA, United States; ^9^The Latino Cancer Institute, San Jose, CA, United States

**Keywords:** breast cancer health disparities, hereditary breast cancer, cancer education, Hispanic/Latina, cancer family history

In the original article, there was an error in **Table 2** as published. The percentages for **Table 2** “Due for Pap. Test (never or 3+ years ago)” for Los Angeles and Sacramento were incorrect. The count and percentage for San Francisco were incorrect. The corrected **Table 2** and its caption appear below. 

**Table 2:** Screening behavior and interest in breast cancer genetics among ‘Tu Historia Cuenta’ study participants (N=1,042) overall and by recruitment area.

Interest and awareness, N (%)	Overall	Los Angeles	Sacramento	San Francisco	P-value
Number of Participants	1,042 (100)	530 (50.9)	264 (25.3)	248 (23.8)	
Interest in learning about genetics and BC					
No Interest	14 (1.3)	11 (2.1)	0 (0.0)	3 (1.2)	<0.001
Some Interest	220 (21.1)	149 (28.1)	23 (8.7)	48 (19.4)	
Very Interested	801 (76.9)	364 (68.7)	240 (90.9)	197(79.4)	
Missing	7 (0.7)	6 (1.1)	1 (0.4)	0 (0.0)	
Genetic Testing Awareness					
Yes	489 (46.9)	224 (42.3)	109 (41.3)	156 (62.9)	<0.001
No	544 (52.2)	297 (56.0)	155 (58.7)	92(37.1)	
Missing	9 (0.9)	9 (1.7)	0 (0.0)	0 (0.0)	
Cancer Screening					
Breast Cancer Screening (Ages 40 to 74)	636	356	160	120	
Up to date with mammogram (<2 years ago)	357 (56.1)	187 (52.5)	97 (60.6)	73 (60.8)	0.200
Due for mammogram (never or 2+ years ago)	272 (42.8)	162 (45.5)	63 (39.4)	47 (39.2)	
Missing	7 (1.1)	7 (2.0)	0 (0.0)	0 (0.0)	
Connected to EWC Program (of those due for mammogram)	64 (23.5)	53 (32.7)	10 (15.9)	1 (2.1)	<0.001
Cervical Cancer Screening (Ages 21 to 65)	1012	512	261	239	
Ever had a pap smear	831 (82.1)	380 (74.2)	233 (89.3)	218 (91.2)	<0.001
Up to date with pap smear	742 (73.3)	332 (64.8)	207 (79.3)	203 (84.9)	0.103
Due for Pap. Test (never or 3+ years ago)	250 (24.7)	165 (32.2)	52 (19.9)	33 (13.8)	
Missing	19 (1.9)	14 (2.7)	2 (0.8)	3 (1.3)	
Colorectal Cancer Screening (Age 50+)	264	158	56	50	
Up to date with colonoscopy	62 (23.5)	33 (20.9)	14 (25.0)	15 (30.0)	0.355
Due for colonoscopy	189 (71.6)	116 (73.4)	42 (75.0)	31 (62.0)	
Missing	13 (4.9)	9 (5.7)	0 (0.0)	4 (8.0)	

Furthermore, there was also an error in **Table 4** as published. The measurement of association should be RRR. An asterisk has also been added to the bottom of the table. The corrected **Table 4** and its caption appear below.

**Table 4:** Multivariate multinomial logistic regression model testing the association between breast cancer screening behavior and demographic factors among ‘Tu Historia Cuenta’ participants ages 40 to 74 (N=587, 42 excluded from 629 due to missing data).

Variable	RRR*	L95%CI	H95%CI	P-value
**Never had mammography (reference)**				
**Mammography up to date**				
Age	1.17	1.12	1.22	<0.001
Years residing in the US	0.98	0.95	1.01	0.175
Immigration status (US born vs. foreign born)	0.95	0.34	2.61	0.917
Educational Attainment (ref: no schooling)				
Elementary	2.56	1.00	6.53	0.050
Middle school	1.54	0.52	4.53	0.435
High school	3.00	1.10	8.20	0.032
Associate degree	2.07	0.66	6.48	0.211
University degree	2.28	0.68	7.62	0.182
Region of residence (ref: Los Angeles)				
Sacramento	2.16	1.23	3.81	0.008
San Francisco	1.08	0.58	2.03	0.801
Insurance Status (ref: no insurance)				
Public	1.58	0.98	2.57	0.062
Private	3.19	1.46	6.98	0.004
English Fluency (ref: monolingual)				
Limited English Use	2.38	1.14	4.99	0.021
Conversational	2.16	1.10	4.25	0.026
Fully Bilingual	1.84	0.69	4.91	0.225
Number of people in the household	0.90	0.78	1.02	0.102
**Mammography more than 2 years ago**				
Age	1.18	1.12	1.25	<0.001
Years in the US	0.97	0.94	1.01	0.118
Immigration status	0.94	0.23	3.79	0.927
Educational Attainment (ref: no schooling)				
Elementary	1.41	0.45	4.48	0.555
Middle school	2.05	0.55	7.63	0.283
High school	2.38	0.69	8.25	0.171
Associate degree	2.43	0.60	9.84	0.214
University degree	2.13	0.48	9.49	0.319
Region of residence (ref: Los Angeles)				
Sacramento	2.33	1.14	4.75	0.020
San Francisco	0.84	0.36	1.94	0.678
Insurance Status (ref: no insurance)				
Public	0.99	0.54	1.84	0.987
Private	1.63	0.61	4.33	0.329
English Fluency (ref: monolingual Spanish)				
Limited English Use	1.60	0.65	3.98	0.307
Conversational	1.18	0.51	2.76	0.694
Fully Bilingual	0.42	0.10	1.71	0.226
Number of people in the household	0.84	0.70	0.99	0.039

*Relative Risk Ratio.

In addition, there were also errors in **Table 5** as published. The sublabels under English Fluency have been updated to match the ones previously used. Furthermore, Insurance status, English fluency and Number of people in household for those with cervical screening 3 years ago were cut off from the table and have been added back in. The corrected **Table 5** and its caption appear below.

**Table 5:** Multivariate multinomial logistic regression model testing the association between cervical cancer screening behavior and demographic factors among ‘Tu Historia Cuenta’ participants ages 21 to 65 (N=932, 80 excluded from 1012 due to missing data).

Variable	RRR*	L95%	H95%	P-Value
Never had cervical cancer screening	Reference			
**Cervical cancer screening up to date**				
Age	1.00	0.98	1.03	0.929
Years residing in the US	1.01	0.99	1.04	0.314
Immigration status (US born vs. foreign born)	0.56	0.26	1.23	0.150
Educational Attainment (ref: no schooling)				
Elementary	1.37	0.64	2.93	0.424
Middle school	1.37	0.56	3.40	0.492
High school	2.03	0.88	4.70	0.099
Associate degree	1.24	0.48	3.20	0.663
University degree	1.31	0.50	3.41	0.583
Region of residence (ref: Los Angeles)				
Sacramento	2.57	1.56	4.22	<0.001
San Francisco	3.71	2.05	6.71	<0.001
Insurance Status (ref: no insurance)				
Public	1.06	0.70	1.62	0.776
Private	3.73	1.67	8.34	0.001
English Fluency (ref: monolingual)				
Limited English Use	1.21	0.65	2.28	0.548
Conversational	1.46	0.83	2.55	0.184
Fully Bilingual	0.64	0.29	1.40	0.264
Number of people in the household	1.06	0.96	1.17	0.259
**Cervical cancer screening 3 years ago**				
Age	1.02	0.98	1.06	0.419
Years residing in the US	1.03	0.99	1.07	0.158
Immigration status (US born vs. foreign born)	0.55	0.17	1.80	0.324
Educational Attainment (ref: no schooling)				
Elementary	1.16	0.30	4.49	0.828
Middle school	2.26	0.51	9.96	0.281
High school	2.82	0.69	11.54	0.150
Associate degree	3.65	0.80	16.73	0.095
University degree	1.41	0.27	7.24	0.683
Region of residence (ref: Los Angeles)				
Sacramento	2.58	1.27	5.26	0.009
San Francisco	3.20	1.32	7.74	0.010
Insurance Status (ref: no insurance)				
Public	0.36	0.19	0.69	0.002
Private	0.77	0.25	2.33	0.638
English Fluency (ref: monolingual)				
Limited English Use	1.25	0.48	3.22	0.645
Conversational	0.86	0.35	2.13	0.747
Fully Bilingual	0.78	0.23	2.57	0.678
Number of people in the household	1.13	0.98	1.30	0.088

*Relative Risk Ratio.

Lastly, there was also an error in **Table 6** as published. The sublabels under English Fluency have been updated to match the ones previously used. The corrected **Table 6** and its caption appear below.

**Table 6:** Multivariate logistic regression model testing the association between colorectal screening behavior and demographic factors among ‘Tu Historia Cuenta’ participants ages 50+ (N=240, 24 excluded from 264 due to missing data).

Never Colonoscopy as reference	OR	L95%	H95%	P-Value
Age	1.04	0.98	1.11	0.165
Years residing in the US	1.00	0.97	1.03	0.882
Immigration status (US born vs. foreign born)	0.98	0.24	3.98	0.980
Educational Attainment (ref: no schooling)				
Less than High School	2.78	1.24	6.47	0.015
High School or more	6.40	2.21	19.37	0.001
Region of residence (ref: Los Angeles)				
Sacramento	1.24	0.52	2.93	0.621
San Francisco	0.99	0.40	2.37	0.974
Insurance Status (ref: no insurance)				
Public	1.24	0.52	2.93	0.621
Private	0.99	0.40	2.37	0.974
English Fluency (ref: monolingual)				
Limited English Use	0.79	0.26	2.54	0.681
Conversational	1.02	0.35	3.14	0.971
Fully Bilingual	1.47	0.39	5.83	0.571
Number of people in the household	0.81	0.66	0.99	0.042

The authors apologize for these errors and state that they do not change the scientific conclusions of the article in any way. The original article has been updated.

